# Supplementary material for: Unveiling the intellectual structure of informality: Insights from the socioeconomic literature
Source: PLoS One. 2024 Jan 31;19(1):e0297577. doi: 10.1371/journal.pone.0297577 (PMC10830049; doi:10.1371/journal.pone.0297577)
Supplement: Attachment — Submitted filename: Response to reviewers.docx [file pone.0297577.s004.docx]

PONE-D-23-06615R1

Unveiling the intellectual structure of informality: Insights from the socioeconomic literature

PLOS ONE

Dear Editor and Reviewers,

We hope this message finds you well. We would like to express our sincere gratitude for your time and commitment to reviewing our article, "Unveiling the Intellectual Structure of Informality: Insights from the Socioeconomic Literature," submitted to the PLoSONE journal. Your valuable comments and suggestions have contributed to enhancing the quality of our work.

We have carefully incorporated the suggested modifications into the manuscript, aligning with your main recommendations. Below, we provide a detailed account of how we have addressed each of the points raised:

| **Comment from editor** | **Answer**  **(The answer to the comment is indicated in green in the manuscript. The description of the modification made (and the page number) is explained below in the column “Answer”)** |
| --- | --- |
| **Comments from reviewer 2** | **Answers** |
| 6. Review Comments to the Author  Reviewer #2: In terms of the methodology, it is imperative for the authors to explicate the rationale behind the selection of the Elsevier Scopus scientific database. The mere mention of its status as the largest curated database worldwide remains insufficient. It prompts the question of the exclusion of the Web of Science. It would be beneficial to explore literature comparing the strengths and weaknesses of both databases, subsequently providing a well-founded justification for the utilization of Scopus. Furthermore, the decision to solely rely on a single database warrants clarification, particularly regarding the transparency and replicability of the research. Elucidating how this approach facilitates ease of study replication by other researchers is imperative. Additionally, an explanation for not utilizing search engines such as Google Scholar is necessary, possibly highlighting the challenges in applying filters due to the inclusion of non-peer-reviewed literature. | We have modified the subsection "2.1. Data Collection" to respond to your comments. Below we present the included text:  This study used academic literature on informality indexed in Elsevier Scopus, one of the largest curated scientific databases in the world (Baas et al., 2020). Scopus data has been extensively used in bibliometric studies and co-word analysis (Zhu & Liu, 2020; REF 2). This database was chosen over alternatives such as Web of Science (WoS) or Google Scholar due to its coverage and academic rigor. Scopus has a broader coverage than WoS, especially in social sciences (Mongeon & Paul-Hus, 2016). Recent research (Singh et al., 2021) indicates that 99.11% of journals indexed in WoS are also included in Scopus. In contrast, only 33.93% of journals indexed in Scopus are found in WoS.  Google Scholar, in turn, has broader coverage than Scopus, but faces significant issues regarding transparency and reproducibility (Gusenbauer & Haddaway, 2020; Waltman & Noyons, 2018). Firstly, it lacks a curated catalog, allowing the inclusion of non-peer-reviewed literature. Furthermore, search results are not consistently reproducible, and it does not support the use of boolean operators. Consequently, thanks to its balance between coverage and rigor, Scopus ensures that our analysis encompasses a broad spectrum of relevant literature (containing most of the WoS content) without compromising on the quality of data.  Additionally, we include 6 new references. |
| Moreover, the incorporation of a research protocol, such as PRISMA, could have potentially strengthened the methodology by facilitating effective data filtration. | Thank you very much for your observation. We seriously considered incorporating the PRISMA protocol into our study. However, since co-word analysis typically involves large volumes of documents, corpus delimitation is usually carried out based on the search query, and therefore, manual or automated filtering and the application of exclusion and inclusion criteria are not used.  In addition, co-occurrence analysis differs from systematic reviews in the goals and reporting standards for results and discussion. PRISMA, in turn, is focused on systematization of results. In contrast, co-occurrence analysis is focused on thematic sense-making.  Finally, the application of the PRISMA protocol is not common, to the best of our knowledge, in studies employing the co-word analysis method. The few examples we identified that integrate the PRISMA protocol in co-word analysis work with relatively small corpus of literature (less than 800 documents. E.g., <https://doi.org/10.3389/fpubh.2023.1075729>, <https://doi.org/10.3390/su13031481>, <https://doi.org/10.14742/ajet.6940>). In our case, the initial corpus was around 3000 documents..  In our case, the methodological framework employed was inspired by the article: Pourhatami A, Kaviyani-Charati M, Kargar B, Baziyad H, Kargar M, Olmeda-Gómez C. Mapping the intellectual structure of the coronavirus field (2000–2020): A co-word analysis. Scientometrics. 2021;126(8):6625–6657. <https://doi.org/10.1007/s11192-021-04038-2> |
| 6. Review Comments to the Author  Reviewer #2: Moving on to the results and discussion, a thorough and coherent presentation significantly contributes to the scientific merit of the work. However, the conclusion could be enhanced by restructuring it into subsections, such as contributions to existing theoretical knowledge, managerial implications, research limitations, and prospects for future research. While this proposed restructuring is not obligatory, it would undoubtedly augment the article's readability and facilitate a more comprehensive grasp of the implications drawn from the findings. | Thanks for this valuable suggestion. We considered this possibility but ended up deciding against it. While potentially useful, we feel it is not possible to satisfactorily flesh out every subsection without changing the current structure and, most importantly, significantly extending the manuscript. As it currently stands, the first three paragraphs of the conclusion are a recapitulation of the results that, we feel, is worthwhile. The rest of the conclusion accommodates, to some extent, prospects for future research. We refrain from forceful expectations or recommendations, given how broad research on socioeconomic informality is and the lack of integration in the communities behind it. At the same time, although some managerial implications could be intuitively derived, such a subsection would mean including some elements of policy and decision-making that the current version of the text lacks. A subsection on limitations would, in turn, require further modifications of the methodology sections, where some of those limitations are currently listed. Finally, a subsection on theory would require a more explicit mention of the current output from the different communities working on socioeconomic informality. |
| 6. Review Comments to the Author  Reviewer #2: In summary, the article exhibits a commendable scholarly effort. However, before final publication, I strongly recommend bolstering the methodology section with a more comprehensive explication of the database selection and an exploration of the justifications underpinning this decision. The remaining recommendations primarily aim to reinforce the existing work, with an emphasis on minor adjustments and refinements. | Thank you for the recommendations. We have adjusted the methodology section to justify the choice of the Scopus database. |

We have worked meticulously on the requested modifications, ensuring that the manuscript is clearer, more rigorous, and valuable to the scientific community. Following the guidelines of the journal, we attach a revised version of the manuscript that incorporates all suggested modifications. In addition, we attach another version in which the revised parts of the document are highlighted to facilitate their identification. We hope these revisions meet your expectations and that the article is now suitable for publication in PLoSONE.

We thank you again for your time and effort in reviewing our work. We value your comments and opinions. We hope that our response and the modifications made are satisfactory. We remain at your disposal for any additional comments or questions you may have.

Kind regards,

The authors
